# Efficacy of Intravitreal Dexamethasone Implant (Ozurdex^®^) in Naïve and Refractory Patients with Different Morphological Subtypes of Diabetic Macular Edema

**DOI:** 10.3390/medicina61030488

**Published:** 2025-03-12

**Authors:** Panagiotis Stavrakas, Evita Evangelia Christou, Vasileios Nasikas, Chrysoula Koutsiouki, Athanasios Vakalis, Solon Asteriadis, Georgios D. Panos, Paris Tranos

**Affiliations:** 1 Department of Ophthalmology, School of Medicine, University of Patras, 26504 Patras, Greece; panos.stavrakas@yahoo.com; 2 Ophthalmica Eye Institute, 54622 Thessaloniki, Greece; info@ocliu-nasikas.com (V.N.); koutsiouki@ophthalmica.gr (C.K.); vakalis@ophthalmica.gr (A.V.); asteriadis@ophthalmica.gr (S.A.); patranos@gmail.com (P.T.); 3 First Department of Ophthalmology, AHEPA University Hospital, School of Medicine, Aristotle University of Thessaloniki, 54636 Thessaloniki, Greece; 4 Division of Ophthalmology and Visual Sciences, School of Medicine, University of Nottingham, Nottingham NG7 2UH, UK

**Keywords:** dexamethasone, ozurdex, macular edema, diabetes

## Abstract

*Background and Objectives*: To investigate anatomical and functional outcomes in different morphological subtypes of diabetic macular edema (DME) treated with an intravitreal dexamethasone implant (Ozurdex) over 6 months follow-up. *Materials and Methods*: A retrospective, comparative study on patients with DME who received an intravitreal dexamethasone implant. Best-corrected visual acuity (BCVA), central subfoveal thickness (CST) and maximum CST on optical coherence tomography (OCT) were measured. The recruits were divided into three groups based on the morphological patterns of DME: serous retinal detachment (SRD), cystic macular edema (CME) and diffuse retinal thickening (DRT). The presence or absence of previous treatment were considered as being previously treated with anti-VEGF (PT) vs. naïve eyes (TN). All subjects received a single injection of the dexamethasone implant. The primary outcomes included changes in BCVA, CST and CSTMax at 2-, 4- and 6-months of follow-up. *Results*: CST was significantly reduced following one dexamethasone injection in the whole cohort from a total mean value of 513.3 μm to 368.2 μm at 2 months, 447.2 μm at 4 months and 471.5 μm at 6 months. The change in CST was significantly greater in SRD as opposed to the DRT and CME group at all time points. Overall, BCVA improved from 0.82 at baseline to 0.75 and 0.76 LogMAR at 2 and 4 months, respectively, whilst showing an overall deterioration to 0.84 at 6 months. The CME group showed the best BCVA at 6 months. Concerning treatment status (TN vs. PT), there was no significant difference in CST at 2 and 4 months, while CST was reduced at 6 months for the PT group (*p* = 0.023). Similarly, BCVA was significantly better in the PT group at 6 months (*p* = 0.017). *Conclusions*: The dexamethasone implant was effective in reducing DME and providing short-term BCVA improvement. The presence of SRD was associated with more favorable anatomical results, while CME was associated with better visual acuity. Dexamethasone provided superior results in previously treated patients.

## 1. Introduction

Diabetes mellitus (DM) is a global health concern with increasing prevalence in the working-age population that substantially affects the quality of life [1,2]. Diabetic macular edema (DME) is the leading cause of severe visual impairment in cases with diabetic retinopathy and may occur at any stage during the course of the disorder [3,4]. The pathophysiological mechanism implicated in DME notably involves the deterioration of the blood–retinal barrier (BRB) that may develop from junctional protein damage and vascular endothelial cell dysfunction, consequently leading to vascular leakage; this may be either focal from dilated capillaries and microaneurysms, or diffuse from retinal vascular structures resulting in fluid accumulation into the macular region [5,6]. In addition, inflammation seems to play a pivotal role in the development of DME; increased levels of inflammatory mediators, such as interleukin-1, -6 and -8, prostaglandins and vascular endothelial growth factor (VEGF) may ultimately lead to the aggregation of extracellular liquid and proteins and, in turn, the distortion of the macular morphology [7,8].

Given that DME may cause irreversible visual loss if left untreated, it has been an unmet need to provide therapeutic options for these patients [9]. Laser photocoagulation had been the gold standard treatment for several years [10]. In an attempt to elucidate the underlying pathogenesis, emerging intravitreal pharmacological therapies have been introduced in the management of DME over the past decades [11]. Anti-angiogenic therapy is based on inhibition of VEGF and is considered to be the first treatment option for DME [12,13,14,15]. In order to reduce the release of inflammatory mediators and inhibit leukostasis, intravitreal corticosteroids are currently used for the treatment of DME. In particular, intravitreal dexamethasone (DEX) implant (Ozurdex, Allergan, Inc., Irvine, CA, USA) slowly releases steroids into the vitreous during a period of 6 months, providing meaningful anatomical and functional outcomes [16,17,18,19].

Spectral domain optical coherence tomography (SD-OCT) provides useful information for identifying the variable parameters of DME, which could serve as predicting factors for the course of the disease or its potential response to treatment [20,21]. Emerging evidence suggests that each morphological subtype of DME may consist of different concentrations of inflammatory cytokines in the aqueous humor [22,23,24]. The latter includes diffuse retinal thickening (DRT), often associated with chronic inflammation and structural damage, cystoid macular edema (CME), typically responding to anti-VEGF therapy but sometimes requiring steroid treatment, and serous retinal detachment (SRD), often linked to high inflammatory activity and benefiting from corticosteroid therapy, which may have a variable response to intravitreal DEX necessitating a tailored therapeutic approach [25,26,27,28].

Given these differences, the choice of treatment must be guided by subtype-specific response patterns. Our study addresses this gap by evaluating the efficacy of dexamethasone (DEX) implants in these subtypes, providing insights into differential anatomical and functional outcomes. We sought to characterize the efficacy of the intravitreal DEX implant to functional and morphological outcomes in various subtypes of DME in treatment-naïve and refractory patients. The aim of this investigation was to identify potentially different responses to DEX that may designate the course of the disease, which undoubtedly affects the quality of life.

## 2. Materials and Methods

### 2.1. Study Design—Participants

This study was conducted at the Ophthalmica Eye Institute between January 2018 and December 2021. The study was approved by the Ophthalmica Eye Institute ethics committee and was conducted in accordance with the principles of the Declaration of Helsinki. Informed consent was obtained from each patient to confirm that their data could be used for research purposes.

This was a retrospective, comparative, single-center study. Consecutive patients aged 18 years or over who met the eligibility criteria were recruited and their data were analyzed. Imaging was performed using SD-OCT (Spectralis—Heidelberg Engineering, Heidelberg, Germany), and the same equipment was used throughout the study. The inclusion criteria consisted of (a) a history of diagnosis of DM type I or II with DME that involved the fovea, without the presence of proliferative diabetic retinopathy; (b) at least one single treatment with the intravitreal DEX implant; (c) a minimum follow-up period of 6 months; and (d) a best-corrected visual acuity (BCVA) score between 90 and 20 letters (Early Treatment Diabetic Retinopathy Study—ETDRS—charts). Both treatment-naïve (TN) and refractory to previous treatment cases were included in the analysis. In refractory or previously treated (PT) patients, anti-VEGF were administered on a pro re nata basis dependent on the persistence of the macular edema; macular edema was defined as a retinal thickness of >250 μm in the central subfield with intraretinal (IRF) or subretinal (SRF) fluid, as seen on SD-OCT. Cases were defined as refractory if they had received at least three consecutive anti-VEGF (ranibizumab or aflibercept) injections applied once a month with no or partial response; the worsening of BCVA by two ETDRS lines; or a reduction of the retinal thickness of less than 10% or a reduction of the central subfield macular thickness of less than 50 μm, as previously described [29]. We excluded cases with macular edema secondary to causes other than DM, namely neovascular age-related macular degeneration, choroidal neovascularization, retinal venous occlusion, uveitis and intraocular surgery within the previous 6 months. Moreover, cases with systemic or ocular conditions that could affect retinal vascular circulation (e.g., a recent cardiovascular event, evidence of macular ischemia defined by fluorescein angiography) and cases with glaucoma or ocular hypertension, dense cataract and the presence of vitreomacular interface abnormalities were excluded from the analysis.

According to the characteristics of DME on SD-OCT images at the baseline visit, the patients were divided into three subgroups: [30] (a) DRT type: a sponge-like retinal swelling of the macula with reduced intraretinal reflectivity; (b) CME type: intraretinal minimally reflective round or oval spaces with highly reflective septa separating cystoid-like cavities that were present in the macular area; and (c) SRD type: the existence of a non-reflective space between the retinal pigment epithelium and the neurosensory retina. As previously described [27], in cases where DRT was combined with CME or SRD, the pattern was appropriately classified as either CME or SRD, and when all subtypes were present (DRT, CME and SRD), the case was classified as SRD. The classification of DME patterns was performed by the same experienced examiner throughout the study. The examiner was masked to the clinical and functional status of the patients while assessing the OCT scans.

The patients were treated with the intravitreal DEX implant according to the standardized protocol. A comprehensive ophthalmological examination, including anterior segment and dilated fundus examination with slit-lamp biomicroscopy, visual acuity and intraocular pressure (IOP) measurement, was performed for each patient at the baseline visit before the injection of the implant and at 2, 4 and 6 months of the follow-up period. At the same time, SD-OCT was obtained for all patients. Re-treatment was considered imperative in cases of CST > 250 μm in the central subfield with the presence of IRF or SRF and reduced BCVA due to recurrent macular edema.

### 2.2. Outcome Measures

The primary outcome measures were the mean changes concerning the BCVA, central subfoveal thickness (CST) and total maximum central subfoveal thickness (CSTmax) between the subgroups of DME in naïve and refractory to treatment patients after the intravitreal DEX implant injection. BCVA was measured using the ETDRS charts. SD-OCT was obtained after pupillary dilation and images were used to evaluate CST and CSTmax, which were automatically generated by the device software. Measurements were recorded at the baseline visit before the DEX implant and at each time point of the follow-up period (2, 4 and 6 months). The secondary outcome measures were the differences between naïve and refractory to treatment patients with DME after the intravitreal DEX implant. Moreover, the IOP elevation during the 6-month follow-up period was evaluated.

In order to increase the reliability of this study, only one eye per patient was considered for the study and for the analysis. This was the first eye of each patient that received the DEX injection, regardless of the status and treatment of the fellow eye. The absolutely necessary follow-up duration for the analysis of primary and secondary outcome measures was set to 6 months’ time, i.e., after the first DEX injection. However, the patients’ data were recorded and further analyzed following multiple DEX injections, always in the same eye.

### 2.3. Statistical Analysis

Statistical analysis was performed using SPSS software (version 20.0; SPSS Inc., Chicago, IL, USA). Continuous variables were reported as mean ± SD for normally distributed data or median [IQR] for non-normally distributed data, while categorical variables were expressed as frequencies (n) and percentages (%). Data distribution was assessed using the Shapiro–Wilk test and visual inspection, and Levene’s test was applied to evaluate the homogeneity of variances. For two-group comparisons, independent Student’s *t*-tests or Welch’s *t*-tests (if variances were unequal) were used for normally distributed data, and Mann–Whitney U tests were applied for non-normally distributed data. For multiple group comparisons, one-way ANOVA with Bonferroni correction or Welch’s ANOVA were used for parametric data, while the Kruskal–Wallis test with pairwise comparisons using Dunn’s test with Bonferroni correction was applied for non-parametric data. Longitudinal changes in BCVA, CST and CSTmax were analyzed using mixed-effects linear models with the patient as the random effect to account for repeated measures over time, as only one eye per patient was included in the analysis. Correlations between the baseline characteristics and outcome measures were assessed using Pearson or Spearman’s correlation, depending on data distribution, and significant associations were further validated using linear regression. Missing data were handled using maximum likelihood estimation within the mixed-effects models, and adjustments for multiple comparisons were made using Bonferroni correction or False Discovery Rate (FDR). Statistical significance was set at *p* < 0.05 (two-tailed).

## 3. Results

A total of 84 eyes of 84 patients were included in the analysis. The baseline demographic and clinical data are shown in Table 1. There were 27 (32.1%) TN eyes and 57 (67.8%) that had received previous treatment with intravitreal anti-VEGF. All patients presented with type II DM. The mean follow-up time was 54.4 months (range 6–159).

The study size needed to achieve power equal to 0.8 with a significance level of 0.05 was 42 eyes, accounting for an expected 2:1 ratio of PT to TN eyes. In effect, the TN group should consist of at least 14 patients, while the PT group should consist of a minimum of 28 patients. We used a two-sided method, and our calculations were based on previous publications that showed a CST difference of 272.0 ± 39.2 and 233.5 ± 65.7 μm in TN and PT eyes, respectively [31].

**Figure 1:**
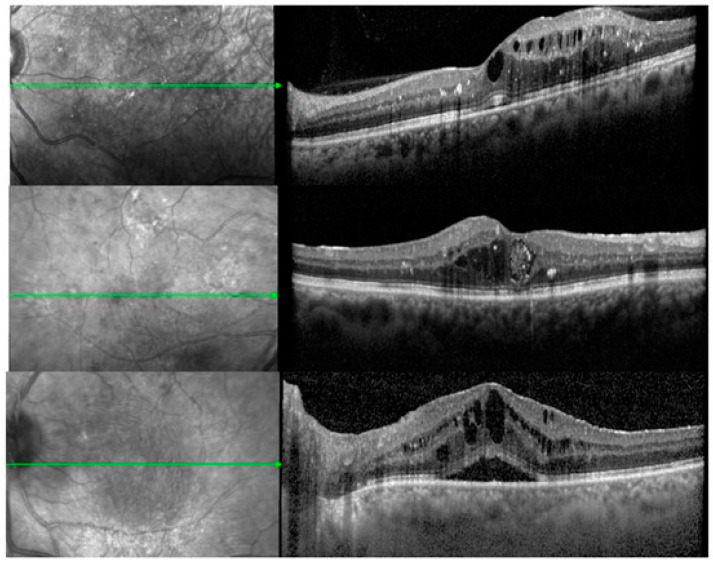
The three morphological types of diabetic macular edema. Diffuse retinal thickening (**upper**), cystoid macular edema (**middle**) and serous retinal detachment (**lower**). The green lines represent scans of the cross sections of the fundus at a specified interval.

Regarding the baseline anatomical characteristics of the DME, as shown on the SD-OCT, 48 (57.1%) had CME-, 15 (17.9%) had DRT- and 21 (25%) had SRD-type. The three morphological types of DME are shown in Figure 1. The mean number of previous anti-VEGF injections in the PT group was 5.1 (SD: 5.7). The total mean CST before the DEX injection was 513.3 μm (SD: 177.8) and the total mean BCVA was 0.8 LogMAR (±0.56) on the EDTRS chart. The IOP before treatment was 14.6 mmHg (SD: 2.7) and no patient was on any antihypertensive topical treatment. The analysis of baseline CST and BCVA between the TN and PT eyes, as well as between the three morphological subgroups, showed no significant differences (TN vs. PT: CST *p* = 0.32, BCVA *p* = 0.94) (CME vs. DRT vs. SRD: CST *p ≤* 0.001, BCVA: 0.84).

### 3.1. OCT Measurements Analysis

**Figure 2:**
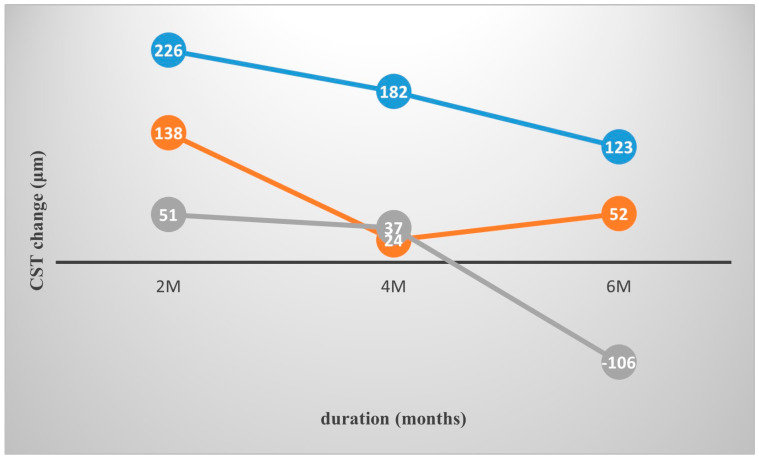
Change in CST (μm) at different time points in the three morphological DME subtypes. Blue line: SRD. Orange line: CME. Gray line: DRT. The reduction in CST was significantly greater in the SRD group as opposed to DRT and CME groups after one DEX injection (*p* = 0.02, *p* = 0.001, *p* < 0.001). Additionally, the DRT group showed deterioration to sub-baseline values.

**Figure 3:**
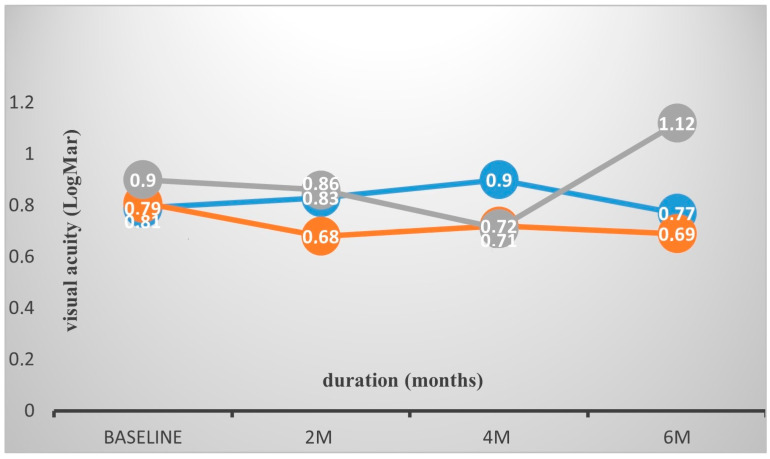
Best-corrected visual acuity (LogMAR) fluctuations at three time points in the three morphological DME subtypes. There was no significant difference between the three groups regarding BCVA at baseline, 2 m and 4 m (*p* = 0.84, *p* = 0.38, *p* = 0.41 respectively). However, the CME group had a significant gain in BCVA at 6 months compared to the rest of cohort.

CST showed significant reduction following one DEX injection in the whole cohort from a total mean value of 513.3 μm at baseline, to 368.2 μm at 2 months post-treatment, 447.2 μm at 4 months and 471.5 μm at 6 months. Similarly, CST was reduced to 405.4 at the final follow-up visit after multiple Ozurdex implants (Table 2). Subgroup analysis of the three types of DME revealed that the change in CST was significantly greater in the SRD group as opposed to the DRT and CME groups for the 2nd, 4th and the 6th month following the first DEX injection (*p* = 0.02, *p* = 0.001, *p* < 0.001). The DRT group was the only group to return to higher than baseline CST values at month 6 after the first DEX injection (difference of −106 μm, SD: 191) (Figure 2). CSTmax was reduced from 583 μm at baseline to 426.6 μm at 2 months, 523.3 μm at 4 months and 558.1 μm at 6 months. Similarly to mean CST, the CSTmax reduction was greater for the SRD group compared to the DRT and CME groups throughout the follow-up period (*p* = 0.006, *p* = 0.0013, *p* < 0.001). As seen before, CSTmax in the DRT group returned to higher than baseline values (difference of −216 μm, SD: 411) (Figure 3).

When the OCT data pre- and post-DEX injection were analyzed based on the treatment status of the cohort (TN vs. PT), there was no significant difference in mean CST at months 2 and 4 (*p* = 0.6 and 0.8, respectively), whereas there was significantly lower CST for the PT group at month 6 (*p* = 0.023) (*t*-test for equality of means). Of note, there was a trend (*p* = 0.051) for the final CST to be lower in PT as opposed to TN after multiple DEX injections. CSTmax and reduction in CSTmax (CSTmax dif) were not statistically different at any time point of the follow-up.

### 3.2. Visual Acuity Analysis

Overall, BCVA improved initially from a mean value of 0.82 LogMAR at baseline to 0.75 and 0.76 LogMAR at 2 and 4 months, respectively, whilst showing an overall deterioration to 0.84 at the 6-month visit (Table 1). There was no significant difference between the three groups regarding VA at baseline, and at 2 and 4 months (one-way ANOVA *p* = 0.84, *p* = 0.38, *p* = 0.41). Nevertheless, VA was significantly better in the CME group as opposed to the DRT group (*p* = 0.05), but not to the SRD group (*p* = 0.193) at the 6-month time point, showing an improvement from 0.81 LogMAR (SD: 0.6) to 0.69 (SD: 0.56). Of note, visual acuity at 6 months, both in the DRT and SRD groups, deteriorated to over the baseline values, from 0.9 (SD 0.3) to 1.12 (SD 0.5) and from 0.79 (SD 0.6) to 0.97 (SD 0.7) LogMAR, respectively.

The visual acuity results were also compared in the TN and PT groups. While there was no difference between the two groups in terms of absolute BCVA values and BCVA difference at months 2 and 4 (*p* < 0.05), BCVA and BCVA difference were significantly better in the PT eyes at month 6 (*p* = 0.017 and *p* = 0.003, respectively). In a similar fashion, the final BCVA after multiple DEX injections at the end of follow-up was statistically better for the PT eyes (*p* = 0.007).

### 3.3. Intraocular Pressure Measurements and Re-Treatment

A significant increase in IOP was observed from 14.6 ± 3 at the baseline to 17.03 ± 3 at 2 months and returned gradually to a mean of 15.6 ± 2.9 mmHg at the final visit. Four eyes (4.8%) with an IOP greater than 23mmHg received topical antihypertensive treatment, which proved to be effective in all cases and no glaucoma surgery was needed in any eye. Seventy-five out of eighty-four eyes required additional DEX implants within the follow-up period. No significant differences in the number of additional injections were observed among the three subtypes of macular edema (*p* = 0.91); however, previously treated individuals required a greater number of implants compared to treatment-naïve patients (3.0 ± 1.4 vs. 3.9 ± 2.7, *p* = 0.05).

Finally, no adverse effects such as endophthalmitis or retinal detachment were noted.

## 4. Discussion

This single-center, retrospective study is one of the few to investigate simultaneously the efficacy of a dexamethasone implant in three morphological subtypes of DME, as well as in TN and PT eyes, with regard to anatomical and functional outcomes. The results showed that Ozurdex was effective in reducing CST in all subtypes of DME (DRT, CME, SRD) at 2- and 4-months following injection, with better anatomical outcomes in the SRD group. There was a decline in the efficacy of the implant in all subgroups towards month 6; thus, a high percentage of eyes (89%) required further DEX injections. On the contrary, visual acuity was found to be better in the CME group and worse in the DRT group. TN and PT eyes showed relatively similar anatomical and functional outcomes for the first 4 months, and surprisingly, PT eyes showed statistically both a higher reduction in CST and better VA at 6 months.

The decline in BCVA in the DRT group suggests that a single DEX injection may not provide sustained functional benefits. Several factors may contribute to this deterioration, such as persistent inflammatory activity leading to recurrent macular thickening, progressive structural damage within the retina, reducing visual potential despite anatomical improvements and possible subclinical macular ischemia, which could limit long-term visual gains. Given these observations, early re-treatment with DEX at four months could be considered to prevent BCVA decline. Prior studies have suggested that DEX implants exhibit peak efficacy between two and four months, with diminishing effects thereafter [17,18]. Implementing a proactive re-injection protocol may help to sustain functional improvements, particularly in patients with chronic DME characterized by DRT.

To date, there is limited evidence concerning the association of DME subtypes, as seen in SD-OCT with the treatment outcomes; in particular, specific morphological characteristics may be considered as prognostic factors of the effectiveness of the applied therapy [20,21]. It is widely known that the underlying pathophysiological mechanism implicated in DME may result from multifactorial pathways; therefore, a number of studies have sought to identify characteristics that are potentially associated with the prognosis or response to various treatment options [3,4]. A well-established feature in the pathogenesis of DME consists of the VEGF [3,4,5,6]. In addition, numerous inflammatory cytokines seem to play a pivotal role in the disruption of the BRB and, in turn, induce vascular permeability and resultant fluid accumulation at the macular area. In fact, increased concentrations of pro-inflammatory molecules, such as interleukin-6, IL-1β, TNFa, intracellular adhesion molecule-1 and monocyte chemotactic protein have been found to mediate the pathophysiology, cause chronic inflammation in the retina and sustain the DME [5,6,7,8]. Based on this mechanism of action, corticosteroids, which downregulate many of the aforementioned inflammatory molecules including VEGF, are used to restore BRB and provide an effective treatment option [19]. Ozurdex intravitreal implant is a biodegradable implant with sustained-released dexamethasone that suppresses DME in the context of inflammation. Currently, it is mainly employed as a second-line therapy for DME in non-responders to anti-VEGF, and is only considered as first-line treatment in patients with specific characteristics (a history of major cardiovascular events, pseudophakic patients) [32].

Our study offers further confirmation that DEX intravitreal implants are an efficacious treatment option in 6-month real-life clinical conditions regarding DME, as shown by previously published studies [25,27,33,34]. However, some of our results come in contrast with recently available data on the same subject.

Concerning the three morphological subtypes of DME, Castro-Navarro and co-authors [34] retrospectively studied 70 eyes, all previously treated with anti-VEGF, and found that although not statistically significant, the anatomical results were better in the SRD group, whereas visual acuity was better in the DRT group. The same authors subsequently studied the three DME subtypes in naïve vs. non-naïve eyes and observed that the type of DME did not have a substantial effect on the treatment outcomes in these two groups of patients [27]. In a previous study by Zur et al. [26] with 299 eyes, retrospective data analysis identified important OCT biomarkers for DME treated with dexamethasone; the presence of SRF, IS/OS continuity and the absence of hyperreflective foci (HRF) could predict better visual outcomes. Additionally, Kaldirim et al. [25], in their series of 35 eyes, found an initially better anatomical response to DEX in the SRD group up to the 4th month, which nevertheless was not sustained up to the 6th month, while showing a significantly better visual acuity in the DRT group at the end of their follow-up. In our study, the data clearly demonstrate that the SRD group had a significantly better anatomical response to DEX at all follow-up time points, further supporting the findings of Navarro et al. [27,34] in PT eyes and those of Zur et al. [26], despite their follow-up concluding at 4 months post-injection. Interestingly and contrary to previous reports [26,27,34], the DRT group in our study, after an initial anatomical improvement, showed a deterioration on all OCT parameters by returning to sub-baseline values at month 6. While the better course of the SRD eyes can be partially explained by the presumed higher inflammatory component in the pathogenesis of subretinal fluid responsive to dexamethasone [22,23,24,25], the deterioration of the DRT group may imply either an even more complex pathogenetic pathway, or a significantly higher inflammatory compound, potentially requiring multiple DEX injections or a combination of treatments from the baseline.

The overall visual acuity results presented herein are in line with evidence reported in clinical trials, such as PLACID [35] and MEAD [36], supporting that the maximum functional effectiveness of the DEX implant happens between the 2nd and 3rd month, while it gradually decreases until 6 months, which is the period in which the macular thickness tends to increase in parallel. Indeed, visual acuity in our study improved significantly at month 2 and 4 while deteriorating at month 6.

Concerning BCVA in each DME subtype, previous studies provide contradictory evidence concerning this topic; the study by Zur et al. [26] with a large cohort found better BCVA results in eyes with SRD after 4 months, while Castro-Navarro et al. [34] did not observe statistical differences between the groups, with a mild trend of better BCVA in the DRT group. Additionally, Kardirim et al. [25] offered evidence from a small group of patients of a markedly better BCVA in the DRT eyes. Our results differ from the aforementioned studies; firstly, the DRT group was the one with the worst BCVA at 6 months. Secondly, the CME eyes surprisingly demonstrated a significant BCVA improvement, although these eyes anatomically (CST and CSTmax) showed a lesser decrease compared to the SRD eyes. Based on the changes in CST and CSTmax, we would expect a trend of BCVA amelioration in the SRD group; however, this was not confirmed. The explanation of these discrepancies can be multifactorial. Our cohort had worse BCVA and higher CST at baseline when compared with the majority of the studies [37,38,39,40,41,42,43], with the exception of the study by Ozsaygili et al. [28] that had a baseline mean CST of 615 μm. It is also known that anatomical outcomes in DME do not always correlate well with functional improvement. Even though direct further comparison with the literature can be problematic due to different study designs, another possible explanation may rely on the presence or lack of presence of specific structural biomarkers such as Disorganization of Retinal Inner Layers (DRILs) and HRF that have been shown to have a robust prognostic factor [26,32,40]. These biomarkers, even in the setting of different subtypes of DME, may alter the treatment outcome [40], a theory which obviously needs to be further elucidated. Our study design was not set to include these OCT biomarkers nor the prevalence of outer nuclear layer (ONL) cysts, which has been found to be equally important [26,40]. However, our results indicate that treatment with DEX can result in a significant improvement of visual acuity even in the CME subtype with poor baseline BCVA.

Recently, studies have focused on the efficacy of the DEX implant for DME in treatment-naïve and refractory to anti-VEGF patients. The IRGREL-DEX study [38], with a large cohort and a follow-up of 24 months, found a strong benefit in both groups following treatment with Ozurdex, but concluded that naïve eyes had significantly better visual acuity and lower CST compared to previously treated eyes. Interestingly, the number of re-treatments was lower in the naïve group as well. The authors suggested that it was the targeting of inflammatory processes and changes in the retinal glia at an earlier stage on behalf of the Ozurdex that might explain the better functional outcomes in treatment-naïve eyes. Zarranz Ventura et al. [41], with cohort mean baseline characteristics similar to ours in terms of BCVA and CST and outside the MEAD criteria [36], found a similar central retinal thickness change in both groups with better BCVA and fewer re-injections in the treatment-naïve eyes. They hypothesized that outer retinal thinning might explain the worst function on the PT eyes. Escobar Barranco et al. [33] showed a mild advantage of the naïve group, which in turn received more subsequent photocoagulations, while noticing that naïve eyes had better baseline BCVA. Similar results in favor of the naïve group were demonstrated by the RELDEX study [19] as well as by Aknin et al. [37] in their small series of 29 eyes. Our results in the TN and PT eyes do present some differences to the ones already published. In fact, CST, CSTmax and BCVA following the DEX injection showed no differences between naïve and recalcitrant cases up to month 4. To our surprise, at month 6, the PT eyes showed significantly better CST, BCVA and differences in BCVA compared to the naïve eyes, in direct contrast to the literature data. Taking into account that the baseline data between the two groups did not markedly differ, the hypothesis for the better results in the PT eyes may once again rely on the specific structural biomarkers of DME. More specifically, at least in our cohort, the “naïve” status of treatment may not necessarily imply a “recent” presentation of DME and prompt treatment. Since this is a real-life retrospective study, some of the naïve treatment patients may have had a neglected DME with HRF or DRILS, precluding them from better visual acuity outcomes. However, in our cohort, the TN group required fewer overall retreatments, which is in accordance with previous studies [38,41]. Furthermore, the percentage of patients with SRD, indicating a relatively recent onset of DME, was only 25%. Overall, the DEX implant considerably improved BCVA, even in the short-term, in both treatment-naïve and previously treated patients, thus providing an additional benefit to the refractory patients.

This study focused on the 6-month results for the primary and secondary outcome analysis. Nonetheless, the recorded follow-up was considerably longer with a mean of 54 months, which allowed us to make additional conclusions regarding re-injections. Indeed, 89% of the patients required multiple DEX injections, a number on the higher end compared to published re-injection rates [18,27,36] and which probably reflects the poor baseline anatomical and functional characteristics of our cohort.

As mentioned by previous studies, the DEX therapy was well tolerated by patients. The main adverse effect was the elevation of IOP, which, in our series, was managed with topical antihypertensive medication. All patients were treated promptly without the need for an antiglaucoma operation in any case. An important consideration when using DEX in clinical care is that there is no existing evidence of a previously cumulative effect of multiple injections on increased IOP, irrespective of pre-existing glaucoma or ocular hypertension [41].

In this study, we evaluated the efficacy of an intravitreal DEX implant in naïve and recalcitrant patients with variable morphological subtypes of DME; the SRD subtype demonstrated significantly reduced macular thickness as compared to CME and DRT. Thus, the presence of the SRD subtype of DME may predict better anatomical outcomes after treatment with the DEX implant. It should be underlined that we analyzed both CST and CSTmax, unlike previous studies in the literature which mainly included central retinal thickness; we indicated that both parameters had the same alteration pattern following treatment. These findings, along with the design of the study, may add strength to our findings. Admittedly, we also recognize certain limitations to our study, including its retrospective nature leading to possible bias, the single-center design and the variability in our study population with different demographic backgrounds. Despite the fact that the sample size was comparable to previous studies, further multi-center larger studies are required to confirm our results and produce more robust evidence. Undoubtedly, the evaluation of structural parameters on OCT such as DRIL, HRF and IS/OS disruption would potentially provide further information for the variable responses of the different subtypes of DME to DEX that may designate the course of the disease. The use of OCTA would add value in assessing macular ischemia, providing further possible explanations for unfavorable functional or anatomical outcomes. In addition, our study did not include data on the duration of diabetes, the diabetic control or other relevant comorbidities such as arterial hypertension, renal disease, lipid profile or HBA1c status, which may have an effect on treatment responses [44,45,46].

Lastly, we acknowledge the fact that lens opacities may have contributed to a relative bias regarding visual acuity, Nevertheless, we have largely counterbalanced this issue by focusing on results obtained up to the 6-month time point, where the progression of cataracts was most probably not significant.

## 5. Conclusions

In summary, the intravitreal DEX implant could provide anatomical and functional improvement in variable subtypes of DME in both treatment-naïve and previously treated patients. In particular, the SRD subtype demonstrated significantly reduced macular thickness compared to CME and DRT, and the CME subtype showed a better functional gain. The DEX injection provided equal and superior results in previously treated patients. Additional prospective longitudinal studies are warranted to validate our findings and determine the accuracy of DME subtype as a predicting factor for the response to DEX implants in clinical practice.

## Figures and Tables

**Table 1:** Baseline demographics of the study population, SD-OCT characteristics and visual acuity measurements in the overall study population, in the DME morphological subgroups and based on treatment status.

Variables	Total N = 84 Eyes	CME Subtype N = 48	DRT Subtype N = 15	SRD Subtype N = 21	TN N = 27	PT N = 57
Age (mean years ± SD, range)	73.1 ± 7.1 (53–86)	74.8 ± 6.0 (62–86)	70.0 ± 11.2 (53–82)	71.4 ± 4.5 (65–79)	72.6 ± 7.3 (62–82)	73.3 ± 7.0 (53–86)
Gender (M/F)	60/24	36/12	6/9	18/3	21/6	39/18
IOP mmHg (SD)	14.6 (2.7)	14.6 (2.9)	16.0 (1.2)	13.7 (2.8)	15.6 (1.07)	14.1 (3.1)
Number of anti-VEGF treatments before DEX, mean (SD)	5.1 (5.7)	4.4 (3.7)	11.6 (8.5)	2.1 (3.4)	-	7.5 (5.5)
CST, mean (SD) μm	513.3 (177.8)	501.3 (93.0)	359.6 (111.3)	650.3 (253.1)	541.2 (245.6)	500.2 (135.3)
CSTmax, mean (SD) μm	583.0 (201.5)	570.7 (89.0)	427.2 (119.6)	722.2 (322.7)	623.1 (301.4)	564.0 (129.8)
BCVA LogMAR (SD)	0.82 (0.5)	0.81 (0.6)	0.90 (0.2)	0.79 (0.6)	0.81 (0.6)	0.82 (0.5)

Abbreviations: CME, cystoid macular edema; DRT, diffuse retinal thickening; SRD, serous retinal detachment; TN, treatment-naïve; PT, previously treated; IOP, intraocular pressure; DEX, dexamethasone; CST, central subfoveal thickness; CSTmax, maximum central subfoveal thickness; and BCVA, best-corrected visual acuity.

**Table 2:** Outcomes of CST, CSTmax and BCVA at three time points (2 months, 4 months and 6 months) following DEX injection, overall and in all study subgroups. Values presented as mean (SD), and CST in μm and BCVA in LogMar.

	Total	CME Subtype	DRT Subtype	SRD Subtype	TN	PT
CST baseline	513.3 (177.8)	501.3 (93.0)	359.6 (111.3)	650.3 (253.1)	541.2 (245.6)	500.2 (135.3)
CST 2 month	368.2 (125.2)	362.5 (109.3)	308.4 (98.4)	424.1 (151.4)	378.5 (119.9)	363.3 (142.2)
CST 4 month	447.2 (139.7)	477.3 (116.2)	322.0 (91.1)	468.4 (168.4)	452.1 (107.0)	445.0 (153.6)
CST 6 month	471.5 (152.4)	448.8 (102.3)	465.8 (98.4)	527.2 (246.2)	525.8 (196.6)	445.7 (119.9)
CSTmax baseline	583.0 (201.5)	570.7 (89.0)	427.2 (119.6)	722.2 (322.7)	623.1 (301.4)	564.0 (129.8)
CSTmax 2 month	426.6 (122.0)	419.9 (113.8)	367.0 (82.8)	484.7 (142.5)	428.5 (93.7)	425.7 (134.1)
CSTmax 4 month	523.3 (131.4)	549.4 (115.2)	395.0 (97.5)	555.7 (135.1)	535.8 (102.6)	517.4 (143.5)
CSTmax 6 month	558.1 (212.7)	511.1 (102.4)	643.2 (199.3)	604.7 (295.6)	608.6 (248.8)	534.2 (191.0)
BCVA baseline	0.82 (0.56)	0.81 (0.60)	0.90 (0.25)	0.79 (0.64)	0.81 (0.64)	0.82 (0.52)
BCVA 2 month	0.75 (0.51)	0.68 (0.56)	0.86 (0.19)	0.83 (0.56)	0.82 (0.56)	0.72 (0.49)
BCVA 4 month	0.76 (0.54)	0.72 (0.60)	0.712 (0.22)	0.90 (0.56)	0.83 (0.57)	0.73 (0.53)
BCVA 6 month	0.84 (0.61)	0.69 (0.56)	1.12 (0.50)	0.97 (0.72)	1.07 (0.70)	0.73 (0.49)

Abbreviations: CME, cystoid macular edema; DRT, diffuse retinal thickening; SRD, central subfoveal thickness; TN, treatment-naïve; PT, previously treated; CST, central subfoveal thickness; CSTmax, maximum central subfoveal thickness; BCVA, best-corrected visual acuity.

## Data Availability

All data are available upon reasonable request. For access to the raw data analyzed in this study, please contact Paris Tranos, Ophthalmica Eye Institute, Thessaloniki, Greece, email: patranos@gmail.com.
